# Four-dimensional computed tomography protocol for preoperative evaluation of the parathyroid glands and its correlations with other imaging methods: a pictorial essay

**DOI:** 10.1590/0100-3984.2020.0056

**Published:** 2021

**Authors:** Stephanie Yuka Matwijszyn Nagano, Almir Galvão Vieira Bitencourt, Ivone do Carmo Gonçalves Torres, Gislaine Cristina Lopes Machado Porto

**Affiliations:** 1 Department of Imaging - A.C.Camargo Cancer Center, São Paulo, SP, Brazil.

**Keywords:** Adenoma/diagnostic imaging, Parathyroid neoplasms/diagnostic imaging, Hyperparathyroidism/surgery, Four-dimensional computed tomography/methods, Adenoma/diagnóstico por imagem, Neoplasias das paratireoides/diagnóstico por imagem, Hiperparatireoidismo/cirurgia, Tomografia computadorizada quadridimensional/métodos

## Abstract

Parathyroid adenoma is the most common cause of primary hyperparathyroidism. Advances in surgical techniques have made it possible to excise only the affected parathyroid gland in most cases. Imaging examinations play a fundamental role in the preoperative planning of parathyroidectomy. To localize the parathyroid glands, imaging tests such as scintigraphy, ultrasound, and, more recently, four-dimensional computed tomography (4D CT). The aim of this pictorial review was to illustrate the use of the 4D CT protocol in cases of parathyroid adenoma and to determine how well it correlates with other imaging methods, in order to improve understanding of the 4D CT method.

## Abstract (pt)

O adenoma de paratireoide é a causa mais comum de hiperparatireoidismo primário. Com o avanço das técnicas cirúrgicas, na maioria das vezes é realizada a retirada apenas da paratireoide afetada. Para que isso seja possível, os exames de imagem têm papel fundamental. São utilizados para a localização das paratireoides exames como a cintilografia e a ultrassonografia, e recentemente a tomografia computadorizada quadridimensional (TC 4D) com protocolo específico. O objetivo deste ensaio é descrever o uso do protocolo TC 4D em casos de adenoma da paratireoide e determinar sua correlação com os outros métodos de imagem, para facilitar o entendimento do método.

## INTRODUCTION

Hyperparathyroidism is a condition caused by an increase in parathormone, a hormone responsible for the release of calcium from tissues into the plasma, either by stimulating osteolytic activity in osteoblasts or by stimulating bone resorption of phosphorus, which contributes to demineralization. There are various types of hyperparathyroidism, which primarily occurs when there is overproduction of parathormone by the parathyroid glands. Parathyroid adenoma is the most common cause of primary hyperthyroidism, accounting for up to 85% of cases, followed by parathyroid hyperplasia, which accounts for up to 12%^(1)^.

The parathyroid glands originate from the third and fourth pharyngeal arches. They are typically located near the posterior surface of the thyroid gland-two near the upper pole and two near the lower pole. Anatomical variations are common, and the parathyroid glands may be in an intrathyroidal location as well as at any site of embryonic development, even in the mediastinum, near the thymus, and although they can be supernumerary, these are rarer^(2)^.

When surgical treatment of hyperparathyroidism began to be implemented, most procedures involved removal of all of the parathyroid glands, because it was not possible to know which gland was affected by adenoma or even to know the precise location of each gland. This procedure was associated with high morbidity, in addition to not always being effective, because anatomical variations and the potential for ectopic parathyroid glands resulted in high rates of recurrence^(2,3)^. Advances in imaging techniques have made it possible to perform minimally invasive techniques, in which the surgeon removes only the affected parathyroid gland, and, with monitoring of clinical parameters and parathormone levels, it is not always necessary to remove the others^(4)^.

**Figure 1:**
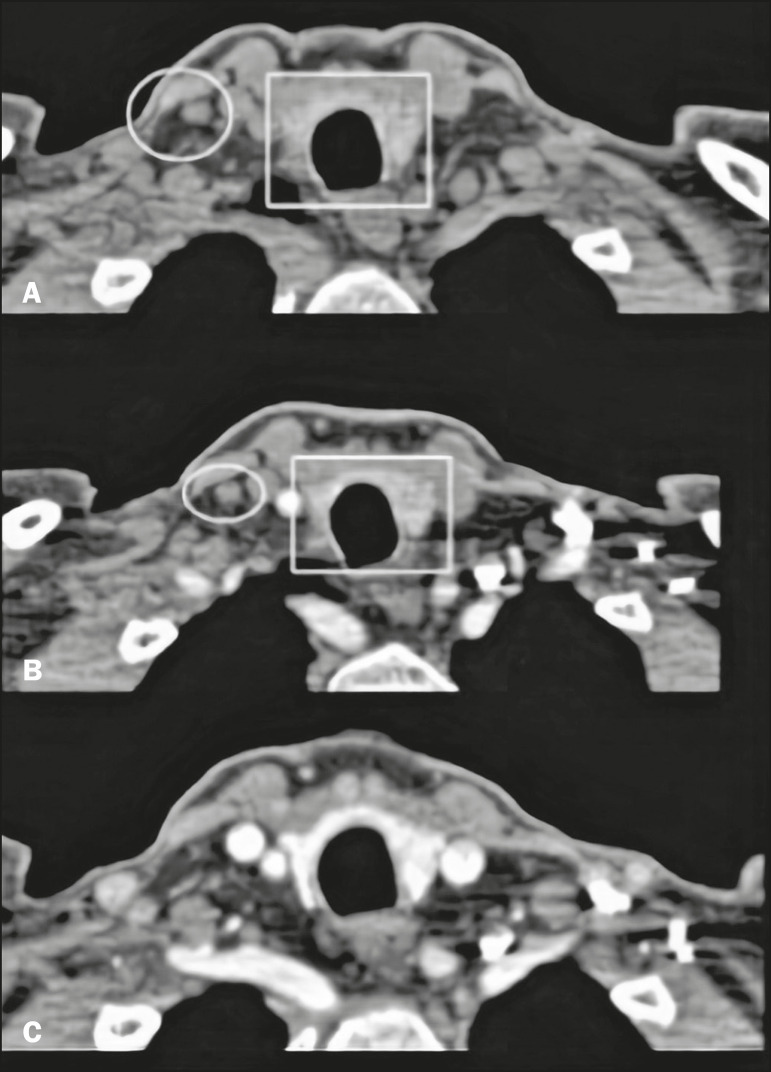
Normal 4D CT protocol. **A**: Unenhanced image showing hyperattenuating thyroid tissue (rectangle) in relation to adjacent lymph node (circle). **B**: Image acquired in the arterial phase, showing the absence of significant enhancement of the nodule (circle) and adjacent thyroid tissue (rectangle). **C**: Image acquired in the venous phase.

Some imaging methods, such as ultrasound and technetium-99m-sestamibi (^99m^Tc-sestamibi) scintigraphy, play a well-established role in the preoperative localization of the parathyroid glands. Four-dimensional computed tomography (4D CT), as illustrated in Figure 1, is a method that was developed more recently and is not yet widely known^(4)^.

The aim of this study was to present a pictorial essay of adenomas, as characterized with the 4D CT protocol, to facilitate the recognition of hyperparathyroidism, as well as to show the correlations that 4D CT has with other imaging methods that play better known roles. To that end, we reviewed the cases of parathyroid adenoma among the case records at our hospital, selecting those in which the 4D CT protocol was used in order to evaluate adenomas and parathyroid hyperplasia, as well as comparable images obtained through other imaging methods.

## DISCUSSION

In the 4D CT protocol for parathyroid evaluation, the third dimension refers to the various reconstruction planes and the fourth dimension refers to time. The 4D CT protocol consists of neck CT with and without intravenous iodinated contrast, including evaluation of the contrast-enhanced images in the arterial and late phases. The unenhanced and contrast-enhanced images are acquired by multidetector CT, the latter being acquired at approximately 25 s and 80 s after contrast injection-in the arterial and late phases, respectively^(5)^.

**Figure 2:**
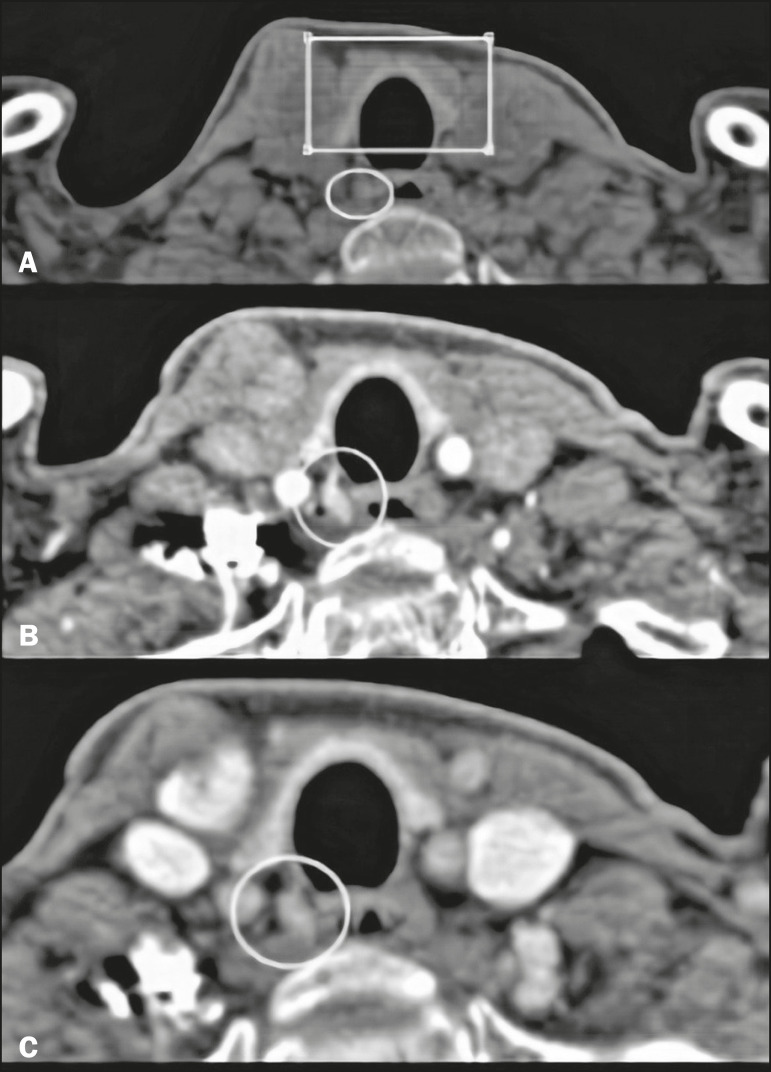
Parathyroid adenoma evaluated with the 4D CT protocol. **A**: Note the area of hypoattenuation (circle) adjacent to the lower third of the thyroid gland (rectangle), which is hyperattenuating in relation to adjacent structures. **B**: Contrast-enhanced image showing avid enhancement of the nodule (circle) in the arterial phase. **C**: Note the contrast wash-out in the late phase (circle).

**Figure 3:**
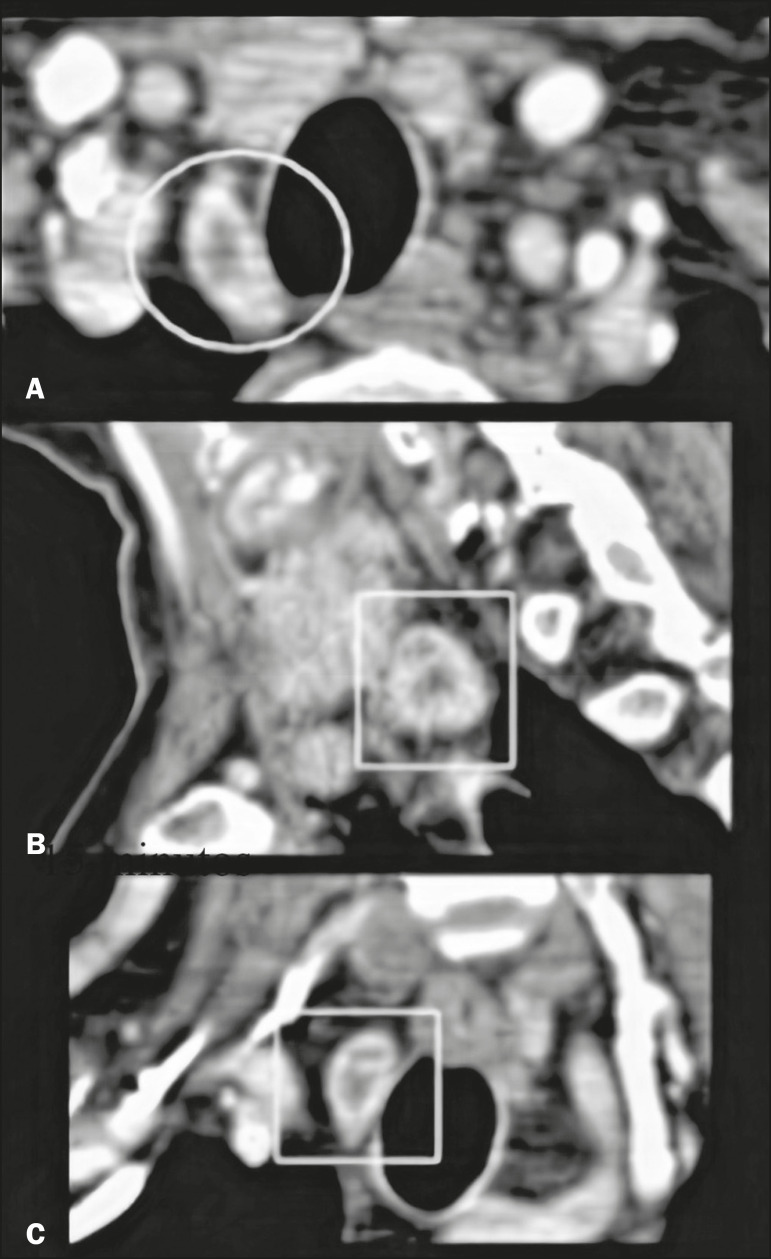
Parathyroid adenoma evaluated with the 4D CT protocol, shown in different reconstructions. **A**: Image of a hyperattenuating lymph node in the arterial phase, showing avid contrast enhancement (circle). In larger lesions such as this one, enhancement is not always homogeneous. **B**: Image showing the nodule (rectangle) in the sagittal plane. **C**: Image showing the nodule (rectangle) in the coronal plane.

**Figure 4:**
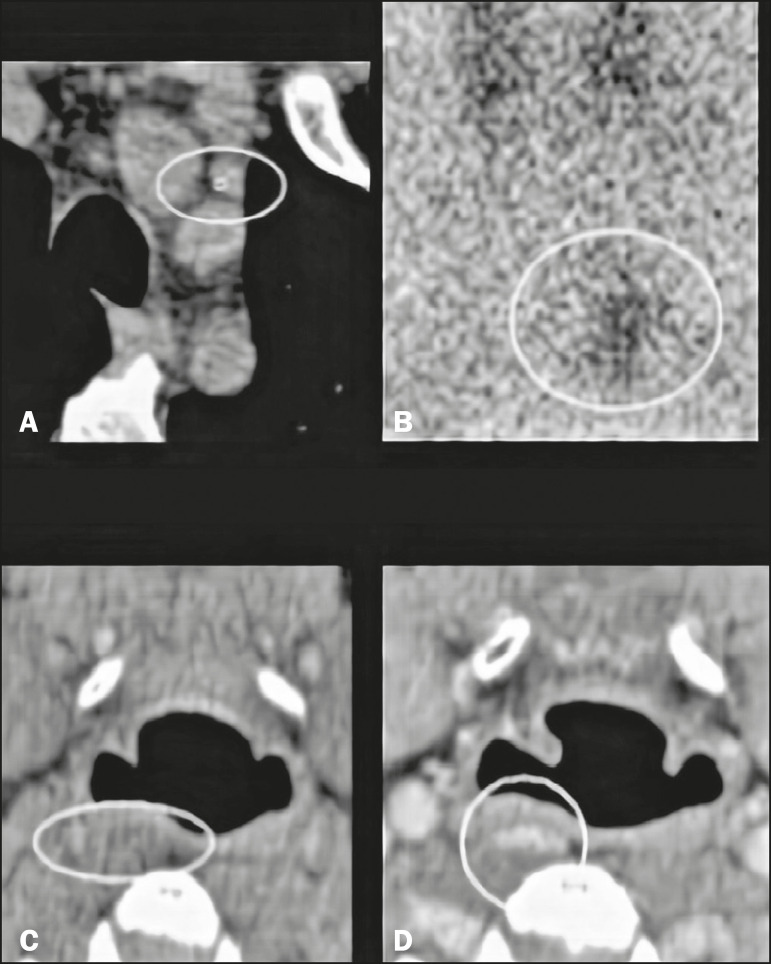
Examples of ectopic parathyroid adenomas. Mediastinal adenoma, adjacent to the aortic arch (circle). **A**: Unenhanced CT scan (in a patient with a history of an allergic reaction to contrast agents). **B**: Correlation of the case shown in A with 99mTc-sestamibi scintigraphy, showing that uptake of the radiopharmaceutical persisted (circle) at two hours after administration. **C**: Unenhanced CT scan of a retropharyngeal adenoma that is difficult to delimit because of the adjacent structures. **D**: Contrast-enhanced CT scan of the case shown in **C**, showing a nodule with intense contrast enhancement (circle). A biopsy of the lesion confirmed the diagnosis of ectopic parathyroid adenoma.

On unenhanced images, the parathyroid glands and the adenomas typically show low attenuation in relation to the thyroid gland. Adenomas, when present, show avid contrast enhancement in the arterial phase and wash-out in the venous phase (Figures 2 and 3, respectively). The study should focus primarily on the arterial phase because it is the most sensitive phase for the identification of adenomas and should consider the other phases only after suspected lesions have been identified^(6)^. The first step is to identify the parathyroid glands in their normal locations, the superior glands typically being located in the upper third of the thyroid gland, whereas the inferior glands are located lateral to, below, or above the lower third. The next step is to look for ectopic parathyroid glands, which can be found from the carotid bifurcation to the mediastinum, according to their embryonic development^(4)^(Figure 4).

The 4D CT protocol is useful not only for determining the location of adenomas, being successful in up to 93% of cases in the preoperative period and in up to 72% of cases in the postoperative period, but also for predicting unfavorable or inconclusive surgical outcomes, as well as for differentiating adenomas from lymph nodes and normal thyroid tissue^(7)^. Lymph nodes exhibit progressive contrast enhancement that peaks in the late phase, whereas thyroid tissue exhibits greater attenuation than does the parathyroid gland on unenhanced images^(7)^. Although 4D CT has good sensitivity, it has some limitations, such as the fact that it exposes patients to higher doses of radiation than does conventional CT and the fact that patient allergy to iodinated contrast is considered a relative contraindication^(8)^.

**Figure 5:**
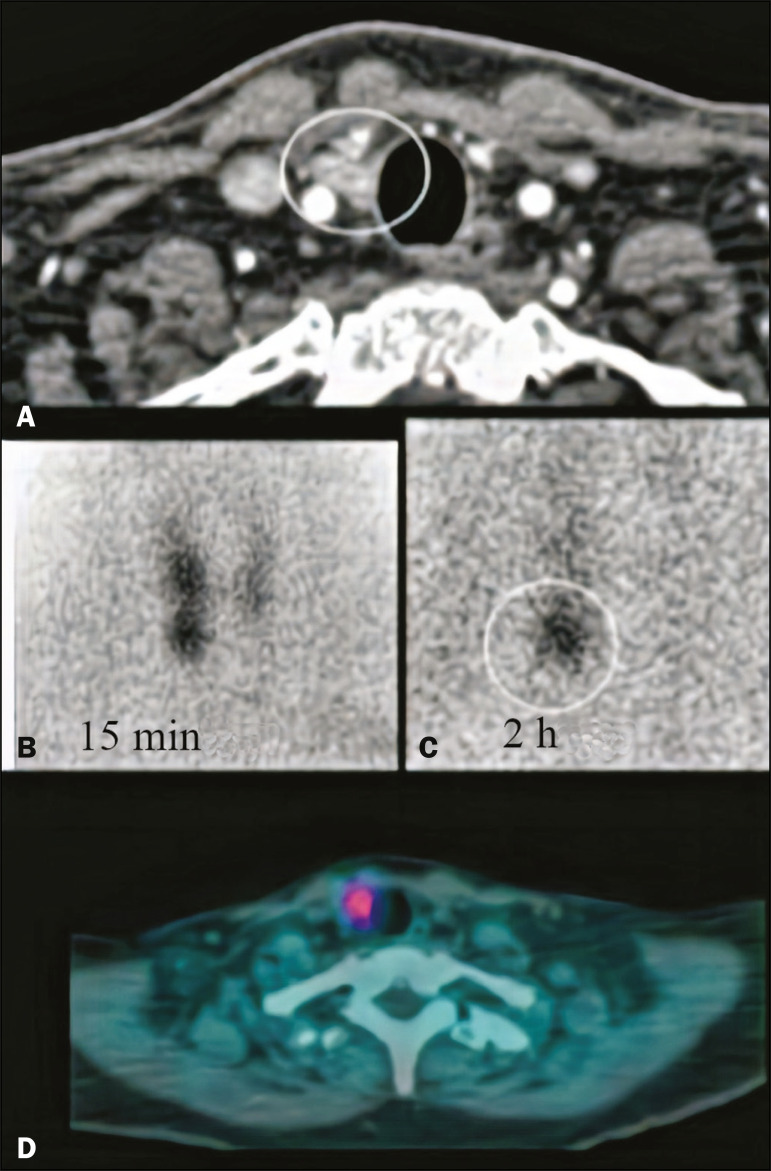
Correlation of 4D CT with 99mTc-sestamibi scintigraphy and with SPECT/CT for evaluation of the parathyroid glands. **A**: 4D CT in the arterial phase showing an area of avid enhancement (circle) adjacent to the common carotid artery and the lower pole of the right thyroid lobe. **B,C**: 99mTc-sestamibi scintigraphy acquired at 15 min and 2 h after administration, showing persistence of the radiotracer adjacent to the lower pole of the right thyroid lobe (circle). **D**: SPECT/CT showing uptake of the radiopharmaceutical (marker) by the adenoma.

Other methods that play a well-defined role in the localization of the parathyroid glands include ^99m^Tc-sestamibi scintigraphy and ultrasound. Planar scintigraphy and single-photon-emission CT (SPECT/CT) are performed with ^99m^Tc-sestamibi, a derivative of isonitrile that was initially used for myocardial imaging and is now also used for parathyroid imaging (Figure 5). In planar scintigraphy, images are acquired at 15-30 min and 2-4 hours after administration of the compound^(9)^. Adenomas are identified by their high metabolic activity. Accuracy ranges from 80% to 95%^(10)^.

**Figure 6:**
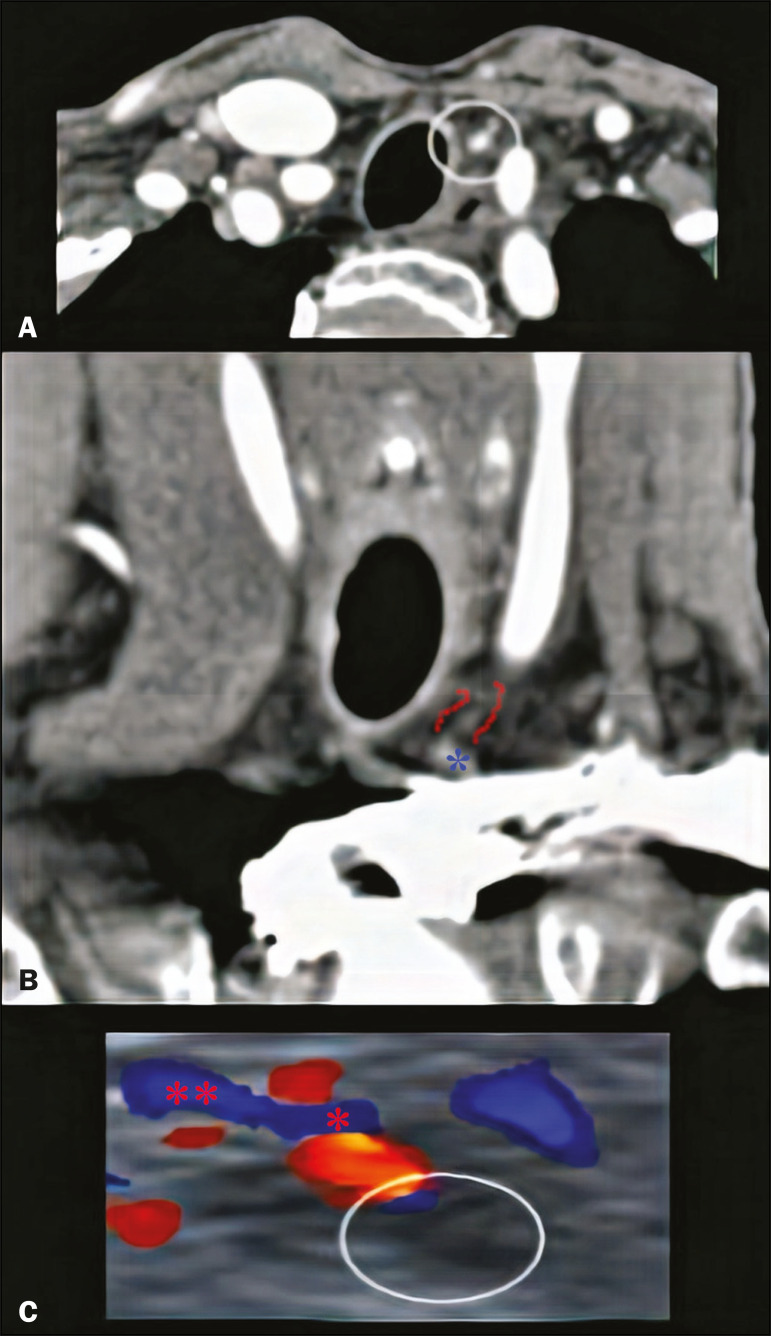
Parathyroid adenoma. **A**: Contrast-enhanced CT scan acquired in the arterial phase, showing a nodule with avid enhancement (circle) in the left thyroid bed. **B**: Contrast-enhanced coronal CT scan acquired in the arterial phase, showing a polar vessel (between the red lines) feeding a nodule with avid enhancement (blue asterisk). **C**: Correlation with ultrasound; color Doppler showing a hypoechoic area in the left thyroid bed (circle), as well as the polar feeding vessel (red asterisks).

Ultrasound was one of the first methods used for evaluation of the parathyroid glands. Although widely used, as a readily available method that does not involve the use of radiation or contrast, ultrasound is operator dependent and thyroid disorders, such as multinodular goiter, may impede the ultrasound evaluation of the parathyroid glands, and it is not an efficient method for the characterization of ectopic parathyroid glands, especially those in the mediastinum. When enlarged, a parathyroid gland is usually seen adjacent to the thyroid gland, with a hypoechoic appearance in relation to the latter. In cases of adenoma, an extrathyroidal polar (feeding) vessel, as depicted in Figure 6, can be observed^(10)^.

Other methods that have been discussed for the evaluation of parathyroid adenomas are dual-energy CT (DECT) and 4D MRI protocol. The DECT method utilizes algorithms to quantify material decomposition and to estimate the chemical composition of an object. This new method plays a role in the localization of parathyroid adenomas by characterizing the attenuation difference between adjacent tissues. For example, it can be difficult to discriminate between an adenoma and normal thyroid tissue in the arterial and venous phases, whereas the two tissue types differ in the degree of attenuation seen on unenhanced images, which makes DECT is a useful tool, especially in cases of intrathyroidal adenoma^(6)^. The 4D MRI protocol is an alternative to 4D CT, because it does not use radiation; however, it is a new method that is not widely available. As in 4D CT, the objective is to explore the hypervascularity of parathyroid lesions, and 4D MRI has high sensitivity for the identification and localization of adenomas-ranging from 92% in single lesions to 77% in multiple lesions^(8)^.

## CONCLUSION

In the evaluation of the parathyroid glands, scintigraphy and ultrasound both play a well-established role and have good sensitivity. The 4D CT method, which, in addition to allowing the diagnosis to be established and adjacent structures to be evaluated, facilitates the differential diagnosis, is still not widely known, despite its high sensitivity. Therefore, it is important for radiologists to be familiar with this novel method.
